# Evidence of Heterosynaptic LTD in the Human Nociceptive System: Superficial Skin Neuromodulation Using a Matrix Electrode Reduces Deep Pain Sensitivity

**DOI:** 10.1371/journal.pone.0107718

**Published:** 2014-09-17

**Authors:** Martin Mücke, Henning Cuhls, Lukas Radbruch, Tobias Weigl, Roman Rolke

**Affiliations:** 1 Department of General Practice and Family Medicine, University Hospital, Bonn, Germany; 2 Department of Palliative Medicine, University Hospital, Bonn, Germany; 3 Department of Anaesthesiology and Intensive Care, University Hospital, Bonn, Germany; 4 Department of Palliative Medicine, Medical Faculty, RWTH Aachen University, Aachen, Germany; University of Texas at Dallas, United States of America

## Abstract

Long term depression (LTD) is a neuronal learning mechanism after low frequency stimulation (LFS). This study compares two types of electrodes (concentric vs. matrix) and stimulation frequencies (4 and 30 Hz) to examine homo- and heterosynaptic effects indirectly depicted from the somatosensory profile of healthy subjects. Both electrodes were compared in a prospective, randomized, controlled cross-over study using 4 Hz as the conditioning LFS compared to 30 Hz (intended sham condition). Quantitative sensory testing (QST) was used to examine 13 thermal and mechanical detection and pain thresholds. Sixteen healthy volunteers (10 women, age 31.0±12.7 years) were examined. Depending on the electrodes and frequencies used a divergent pattern of sensory minus signs occurred. Using LFS the concentric electrode increased thermal thresholds, while the matrix electrode rather increased mechanical including deep pain thresholds. Findings after cutaneous neuromodulation using LFS and a matrix electrode are consistent with the concept of heterosynaptic LTD in the human nociceptive system, where deep pain sensitivity was reduced after superficial stimulation of intraepidermal nerve fibres. Cutaneous neuromodulation using LFS and a matrix electrode may be a useful tool to influence deep pain sensitivity in a variety of chronic pain syndromes.

## Introduction

In contrast to transcutaneous electrical nerve stimulation (TENS) that is a well-known model for treating acute or chronic pain by placing flat gel electrodes over the most painful body area [1], [2], the present study addresses two different types of electrodes for intracutaneous electrical nerve stimulation (IENS). While TENS leads to a diffuse current distribution across superficial and deeper tissues such as the muscle, recent studies have introduced small size concentric electrodes to activate preferentially intraepidermal nociceptive nerve fibres, and to assess cortical excitability after this type of peripheral C- and more important A-delta fibre stimulation [3]–[5]. Different from TENS, the present study compares this type of concentric electrode with a recently developed matrix array electrode, both inducing predominant intracutaneous peripheral input giving rise to learning processes within the upper and deeper layers of the dorsal horn of the spinal cord [6]. In rodent and human models high stimulation frequencies were reported to induce synaptic long term potentiation (LTP) that is characterized by an amplification of synaptic processing resulting in decreased pain thresholds in the presence of increased neuronal responses to repeated stimuli of the same intensity [7], [8]. LTP of the nociceptive system is described to be homo- as well as heterosynaptic, indicating that increased pain sensitivity can be observed within the originally stimulated area as well as in adjacent un-conditioned areas [9]. This effect can be explained at a central level including possible mechanisms such as increase of receptive field sizes of spinal dorsal horn nociceptive neurones, namely wide-dynamic-range neurones (WDR) [10]. In contrast, it is possible to reduce the strength of synaptic performance by applying currents using low frequency stimulation (LFS) [11]. Rodent models were used to assess such a synaptic long term depression (LTD) that is described to be homosynaptic, which corresponds to reduced pain sensitivity at the spot being stimulated before [12]. Based on the literature, we hypothesize a functional sensory deficit (adaptive hypoesthesia and hypoalgesia) within that stimulated skin area across painful and non-painful thermal and mechanical stimuli. In order to assess these changes of sensory percept after LFS we used the standardized quantitative sensory testing (QST) protocol of the German Research Network on Neuropathic Pain (DFNS) to test for the full performance of the somatosensory system [13].

In that context, it is the main goal of the present study to investigate the effects of a recently developed large size matrix array electrode preferentially stimulating superficial layers of the skin in comparison with a concentric electrode to address the following questions: 1) Is it possible to reduce thermal or mechanical perception after LFS? 2) Do both types of electrodes differ from each other, when compared for 4 Hz-LFS and higher stimulation frequencies? 3) Is the effect of LFS restricted to the stimulated superficial skin area (homosynaptic) or even extended to deeper tissues (heterosynaptic) using the larger matrix electrode that allows to stimulate larger skin areas including receptive fields of more spinal WDR-neurones than the smaller concentric electrode?

## Materials and Methods

The trial was a prospective, single-centred randomized, controlled, for the subjects and experimenters completely double-blinded, balanced, crossover study in healthy volunteers. The study design and protocol were reviewed and approved by the Ethics Committee of the Medical Faculty, University of Bonn. The study was in accordance with the ethical principles originating from the Declaration of Helsinki and in compliance with Good Clinical Practice.

### Study population

Sixteen healthy volunteers (6 men and 10 women, age 31.0±12.7 years) were evaluated for this study. The participants received no compensation expense.

#### Inclusion and exclusion criteria

Inclusion criteria were a minimum age of 18 years and a written consent after a detailed explanation of the investigation. Exclusion criteria were contraindications to the use of electrical stimulation such as the presence of cardiac pacemakers or other implanted electronic devices, severe cardiac arrhythmia, osteosyntheses, malignant tumours, neurological diseases, peripheral vascular diseases, pregnancy, breastfeeding women, haemophilia, skin or soft tissue disease or chronic pain syndromes such as migraine or back pain. Contraindications for the application of sensory test method as local or systemic diseases that affected the area to be tested, lead to exclusion. Previous experience with electrical stimulation methods was also an exclusion criterion in order to avoid an expectation bias.

### Materials used

#### Matrix array electrode

**Figure 1:**
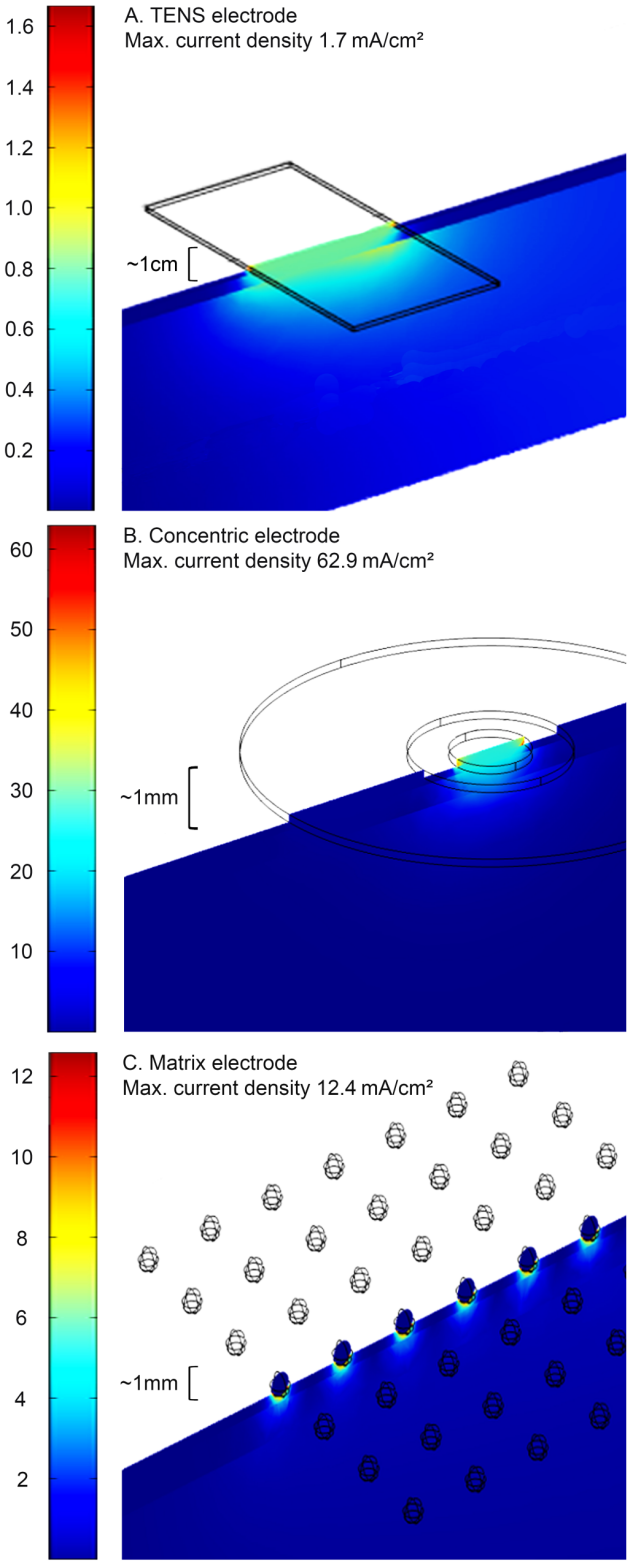
Simulation of the field density of the used electrodes. Using the Finite Element Method (FEM) the distributions of currents are visualized for (**A**) a flat gel electrode that can be used for TENS, (**B**) a concentric electrode, and (**C**) a matrix array electrode. Assuming an ohmic skin resistance and isotropic electrical properties of skin and underlying tissue, only the matrix array and concentric electrodes showed high current densities preferentially distributed across superficial layers of the skin. The gel electrode showed at least 7 times smaller maximum current densities with a much deeper current distribution. Note the different scaling of the colour codings of current densities as well as the penetration depths.

**Figure 2:**
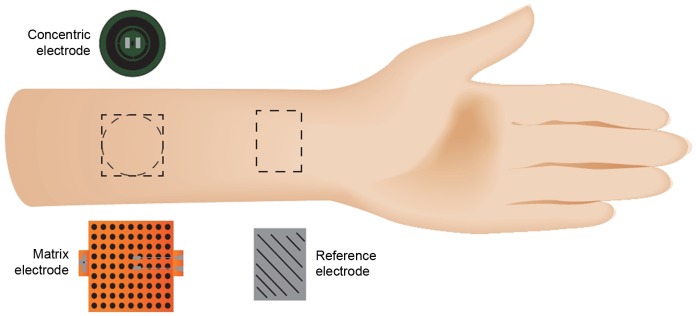
Schematic drawing of stimulation sites at the volar forearm. The concentric electrode serves as its own reference with the cathode centred as a single pin in the centre of the electrode surrounded by the annular reference (anode). The matrix array electrode is used as the cathode with a flat gel electrode that serves as the reference electrode (anode).

The matrix electrode array is a three-dimensional multi-electrode array. By coating it with so-called ball grids (ball grid array), point-wise contact with the skin is ensured (Figs. 1 and 2).

**Figure 3:**
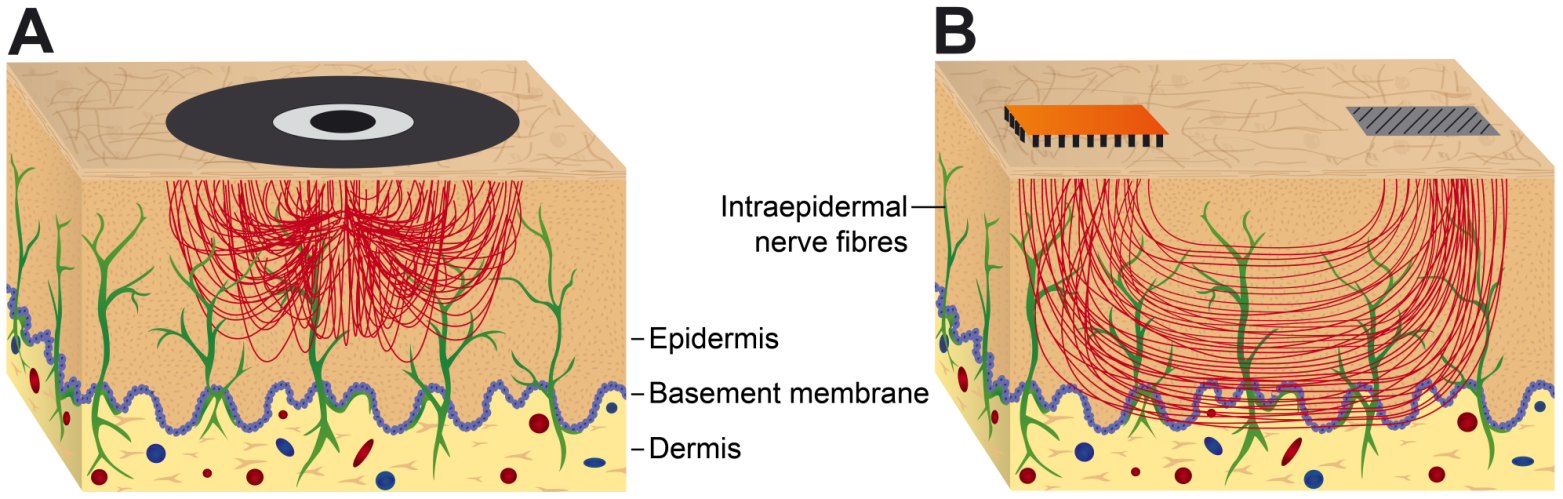
Schematic drawing of the distribution of current density. Both (**A**) a concentric electrode and (**B**) a matrix array electrode induce high current densities distributed within superficial layers of the skin predominately activating intracutaneous nerve fibres.

Individual contact points are each soldered with a spacing of 2.5 mm. The matrix array was used as a cathode and consists of a contact surface with 7 rows and 7 columns, which form a 7×7 = 49 skin contact pin matrix. Using these point-wise small diameter (0.5 mm) skin contacts high electric field densities were reached under each pin. The size of the stimulation area was 40 mm×40 mm (Figs. 2 and 3). A flat 50 mm×50 mm gel electrode served as a reference (anode).

**Concentric electrode.** The concentric electrode was modelled according to published experimental setups [14], [15]. This type of electrode consists of a large annular anode with an inner diameter of 8 mm and an outer diameter of 24 mm. The cathode with a diameter of 1 mm is centred at the middle of the electrode. Due to the concentric design, a high field density is reached under the single cathode pin even with low current densities (Fig. 1). The concentric electrode design allows a preferential stimulation of the superficial layer of the skin. However, the size of the stimulated skin area is significantly low.

#### Stimulator

A constant flow stimulator was used for the cutaneous neuromodulation (model DS7A, Digitimer Ltd., UK). In order to perform frequency-specific stimulation, a trigger unit (model 182A, function generator, WAVETEK, USA) was connected to the DS7A.

### Stimulation paradigms

Based on the literature we used 4 Hz low frequency stimulation over 5 minutes as the conditioning test stimulus [16], [17], and an intermediate 30 Hz frequency as an intended sham frequency [18]–[21]. We have choosen a 4 Hz stimulation paradigm in order to make it more difficult for the subject to distinguish the 4 Hz (in contrast to 1 Hz) conditioning from an intended sham stimulation of intermediate frequencies (30 Hz in the present study). Moreover, other studies have supported the concept that not only the frequency, but also the total number of stimuli applied, plays a role for the established synaptic strength and duration of LTD [20], [22]. Accordingly, the whole amount of stimulation time is reduced, when a 4 times greater number of stimuli per time is applied. Here we were focussing at a total number of 1200 stimuli that could be delivered within the time frame of 5 minutes instead of 20 minutes, when using a 1 Hz stimulation paradigm.

### Quantitative sensory testing (QST)

Seven tests measuring 13 parameters according to the QST protocol of the German Research Network on Neuropathic Pain (DFNS) were used to quantify the performance of somatosensory nervous system. Using this method, properties of nociceptive and non-nociceptive submodalities of different groups of afferent nerve fibres and central pathways are determined. With this test method a complete profile of the somatosensory phenotype can be obtained within one hour. The tests were consistently carried out in the same sequence.

#### Thermal detection, pain thresholds and paradoxical heat sensations

Thermal testing was performed using the Thermal Sensory Analyser II (TSA 2001-II thermal sensory analyser, Medoc, Ltd., Israel). This device is a computer-based system for recording the functionality of thinly myelinated A-delta- and non-myelinated C-fibres. Using a contact thermode the cold detection threshold (CDT), the warm detection threshold (WDT), cold pain thresholds (CPT), and heat pain thresholds (HPT) were determined. The thermal sensory limen procedure (TSL) was carried out for easier detecting of paradoxical heat sensations (PHS). The contact area of the thermode was 9 cm^2^. By pressing a stop button that is connected to a computer unit, thresholds can be determined starting at 32°C with a continuously increasing or decreasing ramp of temperature (1°C/s). The actual thermal detection or pain thresholds were calculated as the mean from three successive threshold determinations.

#### Mechanical detection threshold (MDT)

MDT was determined using a calibrated set of von Frey filaments (Optihair2 set, Marstock nerve test, Germany). These fibreglass filaments of different diameters, but identical texture were all equipped with the same rounded tip with a skin contact area of about 0.5 mm^2^. The set used for testing consists of filaments applying forces between 0.25 and 512 mN with a stepwise increase of force intensities by a factor of 2. MDT was calculated as the geometric mean of five straight above and below-threshold stimulation intensities.

#### Mechanical pain threshold (MPT)

MPT was determined using a set consisting of blunt needles (pinprick, MRC Systems GmbH, Germany) with a skin contact area of 0.25 mm diameter and fixed stimulation intensities between 8 and 512 mN - again with a stepwise increase of force intensities by a factor of 2. MPT was calculated as the geometric mean of five straight above and below pain threshold stimulation intensities.

#### Mechanical pain sensitivity (MPS) and dynamical mechanical allodynia (DMA)

For the determination of MPS and DMA (pain due to light touch), a set consisting of seven pinprick stimulators, a Q-tip, soft brush and a cotton swab were used. This method allows a judgement about the stimulus/response behaviour after supra-threshold pinprick stimulation and primarily non-painful gentle touch. The subjects were asked to rate the perceived pain intensity of the stimuli using a numerical scale with values between 0 and 100 (0 = no pain, 100 = most intense pain imaginable). MPS and DMA were determined within the same test procedure, where each stimulus was repeated 10 times in a completely balanced order over the control and test area. MPT was calculated as the geometric mean of all the individual numerical values after pinprick stimulation. DMA was calculated as the geometric mean of all individual numerical values for light touch stimulators (cotton wisp, Q-tip, brush).

#### Wind-up ratio (WUR)

To determine WUR a needle stimulator with an intensity of always 256 mN was used. The stimulus was applied with an interval of 1 second in a series of 10 needle stimuli. The rating of the perceived pain intensity across the series of stimuli was then compared with the rating to a single stimulus that was applied before starting the series. All stimuli were applied within a skin area of 1 cm^2^. This procedure was repeated five times. WUR was calculated as the ratio of the average pain ratings of the five series of stimuli divided by the mean value of the five individual stimuli.

#### Vibration detection threshold (VDT)

A Rydel-Seiffer tuning fork (64 Hz; 8/8-scale) was used for determining VDT. The vibrating tuning fork was placed over the tested skin area. VDT was assessed as a disappearance threshold by calculating the arithmetic mean of three consecutive measurements.

#### Pressure pain threshold (PPT)

A pressure algometer (Algometer, SBMEDIC Electronics, Solna, Sweden) was used for determining PPT. The pressure algometer has a blunt contact area of 1 cm^2^ to apply a pressure of 0–2000 kPa to the area to be tested. The pressure was increased during the application using a continuously increasing ramp of about 0.5 kg/cm^2^*s (∼50 kPa/s) [13]. The subjects were asked to stop the procedure as soon as the first uncomfortable feeling of pressure occurred. PPT was calculated as the mean threshold of three consecutive measurements.

### Study design

Each subject was randomized prior to testing by picking an envelope, which was immediately transferred to an independent scientific employee, who prepared the predetermined frequencies and types of electrodes as well as afterwards the application of the five-minute stimulation procedure. All stimulations were performed with a matrix array or concentric electrode using 4 Hz as the conditioning test stimulation or 30 Hz as the intended sham stimulation for 5 minutes each. The investigations took place for each subject under identical conditions in a quiet room equipped with a study chair and all study materials. Each subject was tested per test cycle over the contralateral not stimulated forearm (control area) prior to the stimulated (conditioned) test area. It took one hour for performing all measurements at both sides. After the five minute stimulation intervention QST was performed by another examiner (MM) blinded to the conditioning stimulation applied before. QST was used as a psychophysical method directly after but not during the conditioning stimulation.

### Finite Element Method (FEM)

Current distributions across tissues can be calculated and visualized using the Finite Element Method (FEM) [23]. All calculations und illustrations were created using the COMSOL Multiphysics Software (COMSOL Inc., USA). Using this method an ohmic resistance of the skin was assumed. Based on the model the maximum current densities at skin surface were calculated in mA/cm^2^. Moreover, the current distribution assuming isotropic electrical properties across superficial tissue layers was visualized including color coded current densities across these tissue volumes.

### Data evaluation and statistics

#### Evaluation of QST parameters

All QST values except the results of the PHS, CPT, HPT and VDT tests showed a left-skewed, non-parametric distribution and were logarithmically transformed before statistical analysis. The statistical analysis was calculated using Statistica 7.1 software (StatSoft Inc., USA). Differences between the control and test site as well as between the matrix array and concentric electrode were analysed using a repeated measurements ANOVA. Dependent factors were the type of electrode (matrix vs. concentric electrode) and the frequency of stimulation (4 Hz vs. 30 Hz). All QST values of the test area after conditioning stimulation were compared to the contralateral control area without stimulation. Post-hoc comparisons were calculated using the LSD test (least significant difference-test). All data were analysed as an arithmetic average (mean) ± SD of the QST log-data except for PHS, CPT, HPT, and VDT, where raw data were used. Mean values ± SEM (standard error of mean) were used for the graphical representation.

The graphical visualisation of the effects after conditioning stimulation on the somatosensory phenotype was created on the basis of Z values. Here, the log or raw QST values following stimulation were correlated to the contralateral control area using the expression:

Z = (mean test area - mean control area)/SD control area.

#### Factor analysis

Since significant effects on deep pain sensitivity were shown only after the conditioning stimulation using the matrix array electrode, an additional factor analysis is only presented here for the variation of the QST parameters after conditioning stimulation with 4 Hz using this type of electrode. Since the phenomena of PHS and DMA did not occur, the factor analysis was limited to the remaining 11 thermal and mechanical perception and pain thresholds. Only factors with an Eigenvalue >1 were taken into account [24].

## Results

The matrix and concentric electrodes showed a divergent pattern of sensitive minus signs following conditioned stimulation at 4 Hz. According to this trend, the concentric electrode influenced the thermal perception and pain thresholds more, whereas the stimulation using an electrode array led to an increase in mechanical thresholds. These effects were more pronounced after test stimulation at 4 Hz than under the control condition of 30 Hz, which represents an optimum sham frequency only for selected QST parameters such as thermal detection and the pressure pain thresholds. It is worth noting that the deep pain sensitivity was reduced only after stimulation with the matrix array electrode at 4 Hz.

### Susceptibility of the somatosensory phenotype

Healthy subjects showed highly significant changes in the somatosensory phenotype following cathodal LFS. The pattern of sensory changes differed gradually depending on the type of electrode and stimulus frequency used. Mechanical and thermal detection thresholds were increased following the pattern: the thicker the fibre type the more pronounced the adaptive functional response. Mechanical hypoesthesia (MDT, VDT – mediated by large diameter A-beta-fibres) was more profound than thermal hypoesthesia (TSL – mediated by C- and A-delta-fibres). For thermal pain thresholds only the cold pain sensitivity was decreased after concentric electrode stimulation (4 Hz), while heat pain thresholds were unaltered. Mechanical pain thresholds were increased with the exception of the windup-ratio. Interestingly, only after matrix array electrode stimulation with 4 Hz the pressure pain thresholds were significantly increased, while this phenomenon could not be observed using the concentric electrode. The phenomenon of dynamic mechanical allodynia was not detected, neither before nor after conditioning stimulation with either electrode or stimulation frequency, which is in accordance with the observation that this phenomenon is absent in healthy human subjects.

### Analysis of variance (ANOVA)

**Figure 4:**
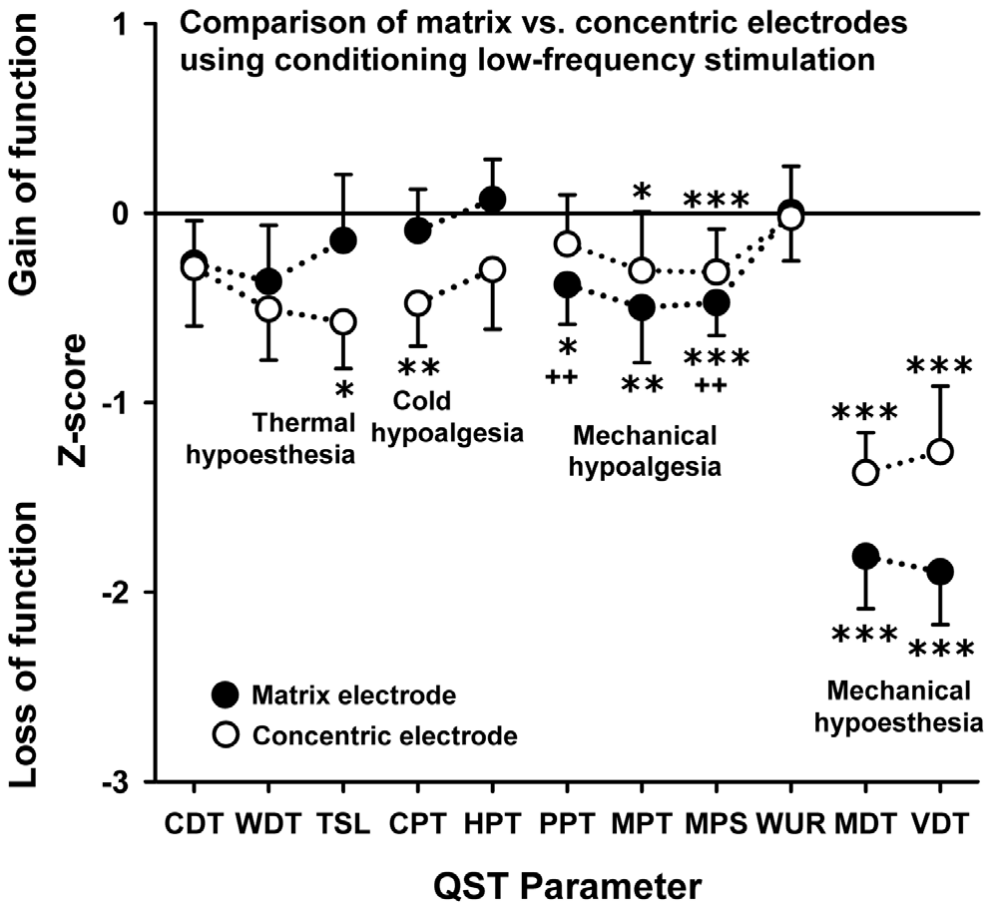
Comparison between the concentric and matrix array electrode after 4 Hz stimulation. Z values are shown according to the expression: Z = (mean baseline - mean stimulation)/SD baseline. A z-value of “0” corresponds to the mean according to the unconditioned control condition. Positive Z values indicate a functional gain, while negative Z values indicate a loss of function for the respective sensory pathway. Stars or crosses denote the level of significance with *p<0.05; **p<0.01; ***p<0.001 for the comparison to the baseline condition; ++p<0.01 for the comparison between electrode types.

**Figure 5:**
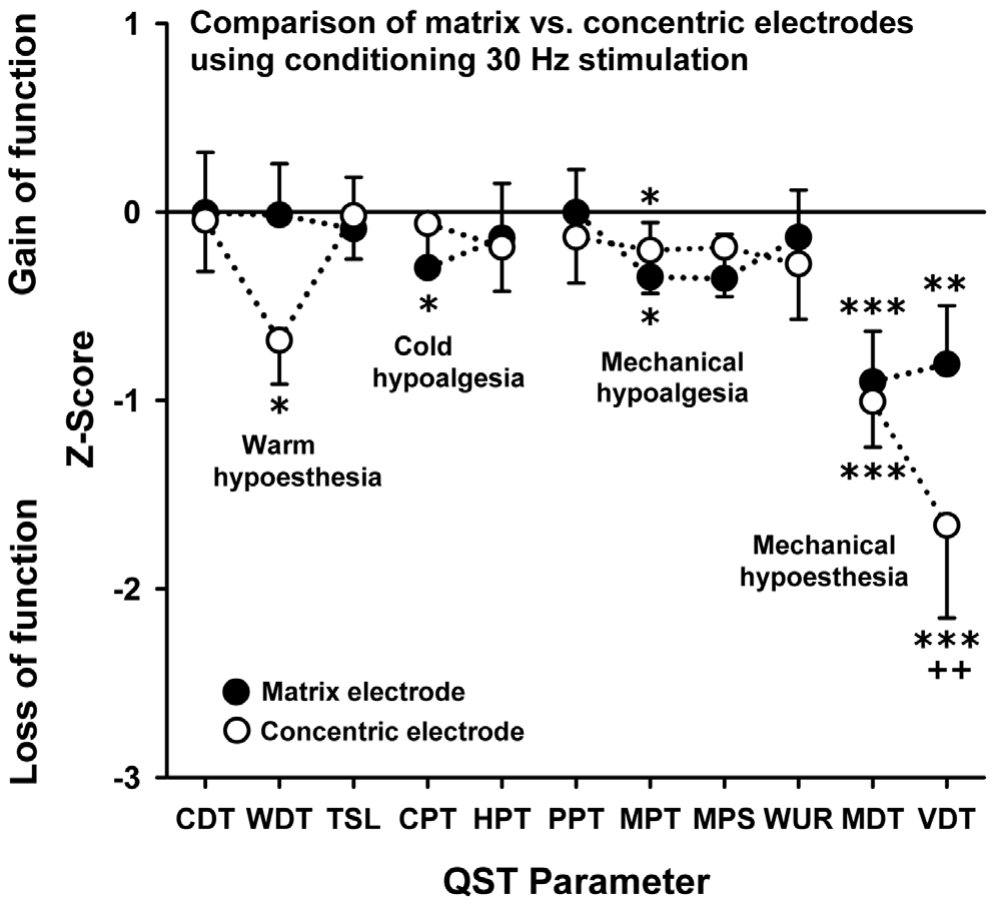
Comparison between the concentric and matrix array electrode after 30 Hz stimulation. The use of this type of a higher stimulation frequency also induced increased thermal and mechanical detection and pain thresholds. Using the matrix electrode thermal detection and deep pain thresholds remained unaltered. Stars or crosses denote the level of significance with *p<0.05; **p<0.01; ***p<0.001 for the comparison to the baseline condition; ++p<0.01 for the comparison between electrode types.

**Figure 6:**
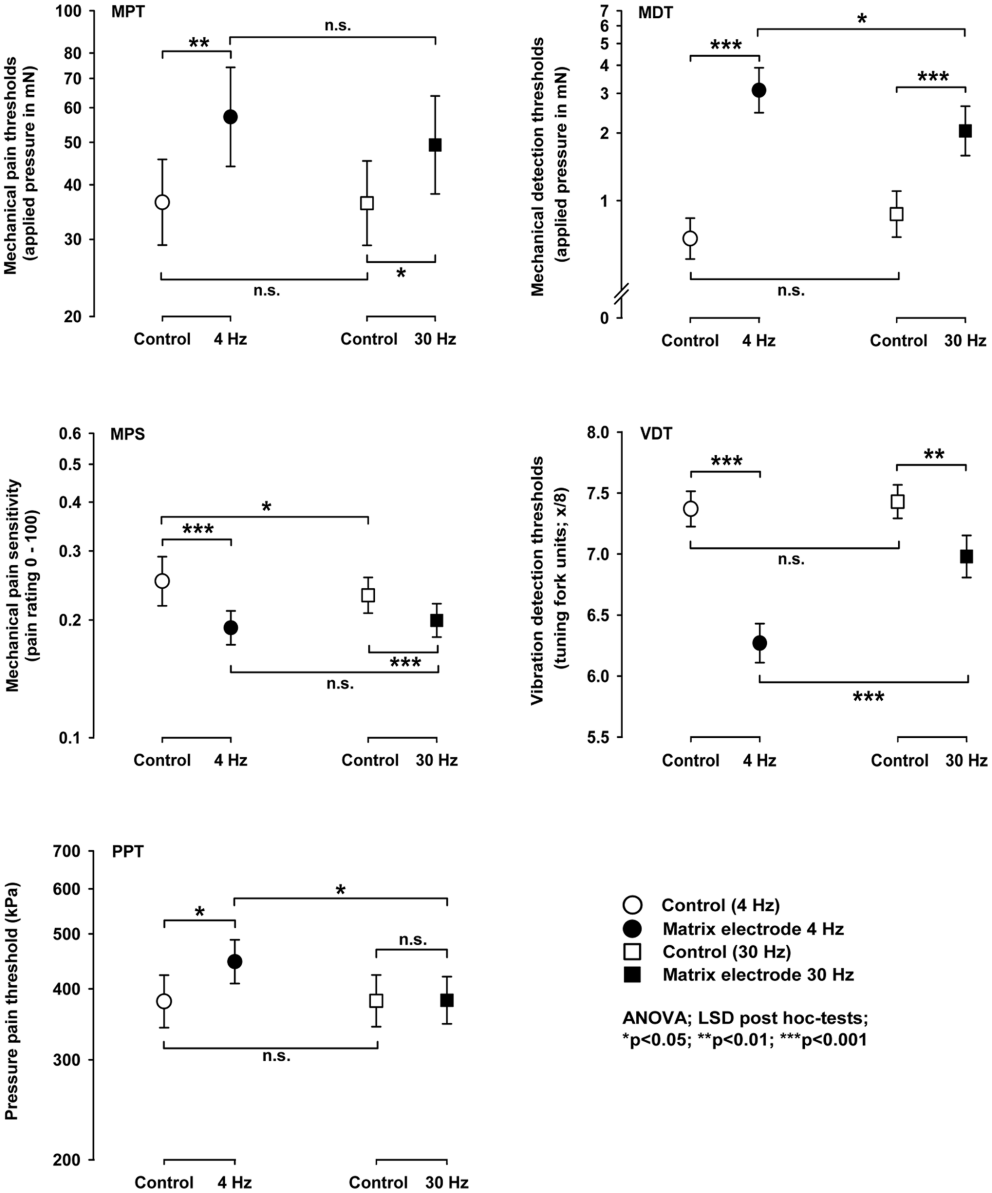
QST raw data compared across different stimulation frequencies. Following conditioning stimulation with the matrix array electrode, all mechanical perception and pain thresholds (except the windup ratio) were significantly increased. This effect was pronounced after 4 Hz rather than 30 Hz stimulation, when compared to baseline condition. Stimulation using a 30 Hz frequency was an optimal sham condition, when assessing deep pain thresholds. However, this frequency still was effective in reducing superficial mechanical sensitivity. Stars or crosses denote the level of significance with *p<0.05; **p<0.01; ***p<0.001 for the comparison to the baseline condition.

As depicted in Table 1 the factor “electrode type” did not show a significant main effect for any of the QST parameters. Also the factor “type of stimulation (4 Hz vs. 30 Hz)” did not show any main affects with the exception of the vibration detection thresholds that represented the most prominently increased QST parameter. The factor “test area” represented the comparison between the unaffected contralateral control and conditioned test area. This factor showed main effects for WDT and CPT, while all other thermal thresholds were not significantly altered. All mechanical detection rather than pain thresholds showed significant main effects with the exception of the windup-ratio. Despite only few interactions of these three factors, there was a significant interaction of the factors “type of electrode and stimulation” for the warm detection thresholds, indicating that only the concentric electrode stimulation significantly increased this measure. A three-way interaction of all factors was found for the vibration detection thresholds, indicating that this threshold was most prominently increased by matrix array stimulation at 4 Hz, while this trend was turned around using 30 Hz stimulation (Table 1, Figs. 4, 5, 6).

**Table 1:** ANOVA of QST parameters.

QST	Factor 1: Type of electrode(matrix vs. concentric)		Factor 2: Type of stimulation(4 Hz vs. 30 Hz)		Factor 3: Test area(stim. vs. control)		Interaction1 by 2		Interaction1 by 3		Interaction2 by 3		Interaction1 by 2 by 3	
Parameter	F-value	P-value	F-value	P-value	F-value	P-value	F-value	P-value	F-value	P-value	F-value	P-value	F-value	P-value
CDT	0.93	n.s.	0.14	n.s.	1.52	n.s.	0.36	n.s.	<0.01	n.s.	0.86	n.s.	0.02	n.s.
WDT	0.01	n.s.	0.53	n.s.	5.77	<0.05	<0.001	n.s.	5.35	<0.05	<0.01	n.s.	1.57	n.s.
TSL	0.01	n.s.	0.81	n.s.	2.11	n.s.	0.41	n.s.	1.35	n.s.	1.3	n.s.	1.33	n.s.
CPT	0.17	n.s.	<0.01	n.s.	8.80	<0.01	2.62	n.s.	0.32	n.s.	0.42	n.s.	5.45	<0.05
HPT	0.83	n.s.	0.32	n.s.	0.98	n.s.	1.81	n.s.	2.58	n.s.	0.05	n.s.	0.40	n.s.
PPT	2.14	n.s.	0.86	n.s.	6.20	<0.05	3.90	n.s.	0.17	n.s.	2.05	n.s.	1.73	n.s.
MPT	0.02	n.s.	3.03	n.s.	15.9	<0.01	1.94	n.s.	0.27	n.s.	0.41	n.s.	0.29	n.s.
MPS	0.09	n.s.	2.45	n.s.	12.4	<0.01	2.20	n.s.	3.05	n.s.	3.27	n.s.	0.25	n.s.
WUR	0.15	n.s.	0.36	n.s.	0.47	n.s.	0.41	n.s.	1.15	n.s.	0.29	n.s.	0.12	n.s.
MDT	0.50	n.s.	1.18	n.s.	63.6	<0.001	0.07	n.s.	0.48	n.s.	1.95	n.s.	2.92	n.s.
VDT	1.53	n.s.	5.08	<0.05	62.1	<0.001	4.17	n.s.	0.74	n.s.	5.44	<0.05	4.79	<0.05

The three-way repeated measurements analysis of variance (F- and P-values) shows highly significant differences comparing the test site after conditioning stimulation and a contralateral un-stimulated control area. Interactions between factors are almost absent; n.s. = not significant.

### Comparison of the matrix array electrode with a concentric electrode

Figure 4 shows a complete QST profile (z values) after conditioning LFS (4 Hz) using both the matrix array and the concentric electrode. The matrix array and the concentric electrode showed a divergent pattern of sensory changes upon stimulation at 4 Hz. Following stimulation with the concentric electrode, the thermal perception and pain thresholds were more increased – at least by trend, while all mechanical detection and pain thresholds were more prominently increased with the matrix electrode. Comparing both types of electrodes, this effect reached significance for PPT and MPS only (LSD post hoc test; p<0.01).

#### Finite Element Method (FEM)

Based on a Finite Element Method (Fig. 1) assuming an ohmic skin resistance and isotropic electrical properties of skin and underlying tissue the highest current densities were reached at skin level using a concentric electrode (62.9 mA/cm^2^) followed by the matrix array electrode (12.4 mA/cm^2^), then a flat gel electrode (1.7 mA/cm^2^) as used for TENS. This finding corresponds with a maximum current density of the concentric electrode at skin level being ∼5 times higher than that of the matrix electrode being ∼7 times higher than that of a flat gel electrode. However, the spatial expansion of the 7×7 = 49 pin matrix electrode (16 cm^2^) was about 3.5 times greater than that of the concentric electrode (∼4.5 cm^2^) with only a single pin centred in the middle of the electrode serving as the cathode. The size of a flat gel electrode as used for TENS corresponded to 40 cm^2^. However, QST experiments were focussing on the comparison between the matrix array and concentric electrode, where the TENS electrode was not investigated.

### Comparing 4 Hz and 30 Hz stimulation

Figure 5 shows the QST profile for both the electrode array and the concentric electrode following stimulation using a 30 Hz frequency. Accordingly, many of the QST parameters investigated, namely the thermal detection and the pressure pain thresholds, did not show significant differences after 30 Hz conditioning stimulation, when compared to the contralateral control site (baseline condition). However, the mechanical pain thresholds to pinprick stimuli were increased for both types of electrodes. Additionally, the cold pain threshold was slightly increased after matrix array stimulation. Interestingly, MDT and VDT were increased using both types of electrode at either stimulation frequency (LSD post hoc-test; all p<0.01). These findings indicate that the intended sham stimulation using a 30 Hz frequency was less effective to reduce mechanical pain sensitivity than the verum stimulation using 4 Hz, while 30 Hz stimulation was similarly effective to increase mechanical detection thresholds pointing to a differential modulation of the different sensory pathways involved.

### Factor analysis of QST data after 4 Hz matrix array electrode stimulation

**Figure 7:**
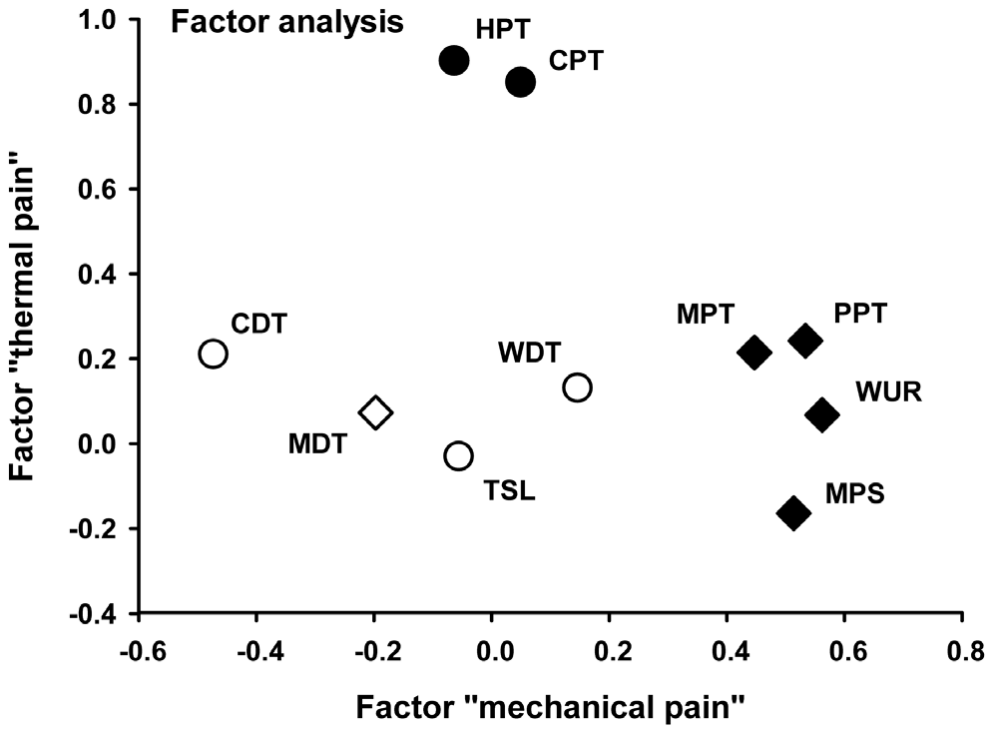
Factor analysis of VARIMAX rotated QST data after 4 Hz matrix array stimulation. All mechanical pain thresholds (MPT, MPS, WUR, PPT) show significant loadings on factor 1 “mechanical pain” (x-axis). Thermal pain thresholds show significant loadings on factor 2 “thermal pain” (y-axis), while thermal and mechanical detection thresholds take an intermediate position, indicating that 4 Hz stimulation differentially effects different peripheral and central sensory channels to the brain.

A principal component factorial analysis was computed using varimax rotated QST data. Using a four-factorial model with factor 1 representing “mechanical pain”, factor 2 “thermal pain”, factor 3 “mechanical perception”, and factor 4 ”thermal detection” altogether 78.5% of the total variance after low frequency matrix stimulation was explained. This statistical approach allows judging the influence of this type of cutaneous neuromodulation on different sensory pathways by comparing factor loadings. Figure 7 illustrates the different factor loadings of all relevant QST parameters on factor “thermal pain” (y-axis) and factor “mechanical pain” (x-axis). The more continuous distribution of explained variance across all 4 factors indicates that 4 Hz stimulation alters processing of all types of sensory submodalities (Table 2).

**Table 2:** Factor analysis of QST parameters.

	Factor 1– Mechanical pain	Factor 2 Thermal pain	Factor 3 Mechanical percept	Factor 4 Thermal percept
**CDT**	−0.27	0.21	−0.08	0.87
**WDT**	0.35	0.13	0.26	0.79*
**TSL**	0.14	−0.03	0.11	0.94*
**CPT**	0.25	0.85*	−0.18	0.13
**HPT**	0.14	0.90*	0.32	−0.03
**MDT**	0.00	−0.07	−0.92*	−0.13
**MPT**	0.65#	0.21	−0.32	−0.16
**MPS**	0.71*	−0.16	−0.04	0.33
**WUR**	0.76*	0.07	−0.03	0.00
**VDT**	0.36	−0.77*	0.05	−0.24
**PPT**	0.73*	0.24	0.36	0.11
**Explained variance %**	22.5	21.3	11. 9	22.9

Varimax rotation of QST data after 4 Hz stimulation using the matrix array electrode.

*Factor loadings >0.70, #factor loadings <0.70, but difference to alternative factors >0.50.

## Discussion

The observed reduction of deep pain sensitivity after a 4-Hz cutaneous neuromodulation using a matrix array electrode is consistent with the concept of heterosynaptic LTD of the human nociceptive system. This concept involves a reduction of pain sensitivity mediated within the central nervous system that has been described before [15], [25]. A direct effect to deeper tissues seems unlikely, since in our model current densities are mainly distributed across superficial layers of the skin using a matrix array electrode (Fig. 1), where small diameter sensory nerve fibres mediate this type of low frequency stimulation to dorsal horn neurones of the spinal cord. However, this spinal site still is the black box of neuronal interactions - depressing or facilitating processing of nociceptive and non-harmful peripheral input that may interact or not while being split to divergent sensory pathways to the brain.

### Changes of the somatosensory profile after low-frequency stimulation

Most prominently the mechanical detection thresholds to von Frey-filaments and a vibrating tuning fork were increased. This finding was more or less independent from the type of electrode or the stimulation frequency used, indicating that these A-beta fibre-mediated stimuli were consistently processed, when the stimulation area was smaller or larger and within a wider spectrum of lower stimulation frequencies, here in the range between 4 Hz and 30 Hz. A-beta fibre input in general is mediated in the dorsal horn of the spinal cord via projection neurones that are different to nociceptive wide-dynamic-range neurones (WDR-neurones). However, recent models of central sensitization explain this phenomenon considering direct or indirect input via interneurons to these WDR-neurones [26]. Possibly, low frequency stimulation within the range between 4 and 30 Hz may inhibit these direct or indirect A-beta fibre projections or activates inhibitory interneuron functions at this or a more rostral level of the CNS. The effects observed were on group level close to a z-score of “2″ corresponding to the upper limit of a 95% confidence range. Less prominent, mechanical pain thresholds were significantly increased following 4 Hz matrix rather than concentric electrode stimulation. This effect was almost absent after 30 Hz conditioning stimulation, indicating that mechanical pain sensitivity mediated is more selectively influenced than mechanical perception of non-harmful stimuli. These interesting findings point to a different susceptibility of spinothalamic (pain) vs. lemniscal (touch, vibration) projection pathways to LFS compared to the spinal or more rostral processing of non-painful tactile input. Importantly, only the matrix array electrode reduced mechanical pain sensitivity for acute, superficial, but also deep pain. In order to evaluate the influence of skin sensory nerve fibres when assessing deep pain thresholds, twelve additional subjects were tested non-blinded using local anaesthesia (5% EMLA cream applied topically for 60 min.) over the volar forearm directly after 4 Hz matrix stimulation. This test again demonstrated an increase of deep pain thresholds by 24.7% (p<0.01, paired t-test) after 4 Hz matrix stimulation when compared to the contralateral control arm. In the present study without exposure to EMLA the increase of the pressure pain threshold was 17.4% (p<0.05, paired t-test). The difference of about 7.3% additional increase after EMLA may represent the contribution of skin sensitivity to that deep pain threshold. This finding is consistent with other PPT data after EMLA cream showing that skin sensitivity explains up to 9.0% of deep pain thresholds [27]. This finding indicates that indeed deep pain sensitivity is increased and that this phenomenon cannot be explained as an epiphenomenon of altered skin sensitivity. One approach to explain this can be sought in a central inhibition of the pain processing system. The central mechanisms addressed by the LFS are not completely understood yet. Thus, the threshold shift shown may originate both from the level of the dorsal posterior horn as well as higher sensory centres, such as the thalamus or the somatosensory cortex [28]. Ex-vivo studies on spinal cord sections showed that the superficial layers of the spinal cord, in particular lamina I spinobrachial projection neurons, play a role in the activation of cutaneous afferents [29]. It could be demonstrated for the nociceptive system that LTP leads to a secondary mechanical hyperalgesia [8], [9]. LTP may be pronounced both homosynaptically with a hyperalgesia at the site of stimulation or heterosynaptically with hyperalgesia in adjacent areas of the skin [8]. Similar processes can be assumed for LTD [4]. Prior evidence for heterosynaptic LTD was reported for the human trigeminal system, where noxious LFS applied to skin afferents of the contralateral forehead via a concentric electrode significantly reduced the blink reflex (heterotopic effect), whereas pain sensitivity was decreased only after homotopic LFS [30]. In the present study we extend this finding of heterotopic LTD to deeper tissues after superficial skin stimulation.

Additionally, thermal hypoesthesia (TSL after 4 Hz; WDT after 30 Hz stimulation) was observed using the concentric electrode only. The similar phenomenon of a tactile hypoesthesia was observed following intradermal injections of capsaicin [31]. Obviously, different sub-modalities appear to be crucial for this mechanism - as hypoesthesia occurs in association with either a hypoalgesia (as shown here after 4-Hz conditioning stimulation) or hyperalgesia [28]. It is likely that different interneurons (GABAergic, glycinergic, opioidergic) control pre-synaptic or post-synaptic functions and are dependent on the frequency and intensity of the stimulus [32], [33]. In various surrogate models it has already been shown that painful LFS leads to LTD with signs of hypoesthesia [4], [8], [11], [34]. It has also been reported that LTD can be induced by high frequency stimulation. However, this requires heterosynaptic processes in which interneurons reduce their discharge frequency [28]. Another consideration would be that LFS leads to an LTP of inhibitory interneurons. This possible mechanism has not been studied experimentally yet. Central mechanisms may also contribute to the triggering of LTD. Thus, it could be shown, for instance, that the processing of afferent tactile stimuli in the 3b site of the cortical S1 region may be centrally modulated by painful stimulation [35]–[37]. Further studies with functional magnetic resonance imaging showed that central deactivations occur in the S1 and S2 regions during stimulation [38], [39]. Probably, a combination of brain and spinal mechanisms may be responsible for the development of LTD. Moreover, either type of electrode was able to induce cold hypoalgesia depending on the stimulation frequency used, a phenomenon that is discussed to be of peripheral and/or central origin. However, the absence of any heat hyperalgesia, which is the cardinal sign of a localized peripheral sensitization, points to a centrally mediated cold hypoalgesia after conditioning LFS.

### Differential effects of matrix array vs. concentric electrode stimulation used for a preferential IENS

A concentric electrode and a matrix array electrode were compared that differ primarily in the arrangement of the anode, cathode and spatial distribution of the stimulation area. For the concentric electrode, the spacing between cathode and anode was only 2.5 mm, so that the penetration depth of the electrical current in the skin was small [14]. For the matrix electrode array, the spacing of 5 cm between cathode and anode was greater. Due to the point-wise contact of the cathode matrix surface, high local current densities occur that are primarily distributed within the upper skin layers (IENS; Fig. 3). This approach of the present study is different to large-surface gel electrodes that are used for TENS, where in contrast to the matrix array electrode a flat and more profuse skin contact exists. This flat type of skin contact results in a more profuse tissue stimulation with lower currents per skin tissue volume. Since nociceptive free nerve endings are found in the superficial layers of the skin [40], they can be more effectively stimulated by a matrix or concentric electrode.

Using a 4 Hz conditioning stimulation the matrix array and concentric electrode show different effects on mechanical and thermal thresholds. Generally, the matrix array electrode increases mechanical rather than thermal thresholds, while the inverse effect was found after concentric electrode application. The electrode design seems to be crucial in this aspect. It seems that varying arrangements and modes of skin contact of the cathode and anode including the resulting different current densities can differentially influence nociceptive fibres. A change in the performance of the pain processing nervous system was shown in previous studies with the concentric electrode, when compared to conventional electrodes [14], [15]. Calculations of the field density of the used electrodes (Fig. 1), based specifically on the Finite Element Method (FEM), showed that the matrix array electrode might induce higher cutaneous current densities than, e.g., a gel electrode. Hence, the triggering of action potentials originating from intracutaneous nerve fibres is more likely to be induced by a matrix or concentric rather than a gel electrode. Moreover, the current density decreases strongly with higher penetration depth based on the FEM model. It is also likely that the point-wise contact area of the matrix electrode array and the greater anode spacing may be beneficial for selectively affecting mechanical thresholds. The electrode placement influences the flow of current, which in turn has a corresponding effect on the activation of cutaneous afferents. For the concentric electrode, the spacing between the anode and cathode is only a few millimetres resulting in high-density currents of superficial skin layers within a small area being stimulated. This design may be important for a stimulation that includes a small spatial distribution across the receptive field areas of only a few spinal WDR-neurones that are important for intensity coding of a peripheral sensory input. Based on the literature, such receptive field sizes can be assumed to correspond to areas of about 1.3 to 1.5 cm^2^ in the volar forearm [41]. Applying currents using our concentric electrode (∼4.5 cm^2^) a maximum of about 4 receptive field areas can be covered, when the electrode is ideally placed over a spot, where these receptive fields border each other. Assuming a model, where these receptive fields are square-wise placed, the larger matrix array electrode (16 cm^2^) covers at least 9 or more receptive fields. This factor is possibly crucial to understand the differences between the concentric and the matrix array electrode. Hence, most likely the matrix array is able to activate much more WDR-neurones per stimulus than the concentric electrode. Moreover, a greater clinical relevance may be attributed to the matrix array electrode, since the modulation of mechanical pain thresholds allow targeted therapeutic approaches. In particular, the triggering of a stable mechanical hypoalgesia including pressure pain thresholds suggests that even pain syndromes of deeper tissues may be treatable. The influence of thermal perception and pain thresholds may be of little clinical significance, for example, potentially in the tumour treatment of patients with cold hyperalgesia following oxaliplatin therapy [42]. Further studies with modified electrode models with optimised configurations may allow more detailed insights into their mechanism of action and the spatial distribution of therapeutic action.

### Does frequency matter?

Low frequencies are suitable for the stimulation, in particular, to trigger synaptic LTD [4], [22]. At these low frequencies using monopolar pulses, the capacitive component of the skin can be disregarded. Therefore, a purely ohmic resistance can be assumed in the Finite Element simulation. [43]. The cathode area may also play a crucial role in triggering LTD. Thus, it is likely that the largest possible area of a receptive field must be stimulated to optimally induce LTD, since a minimum number of afferents must be stimulated first to trigger this mechanism [44]. Based on the literature [18]–[20] it was hypothesised that a 30-Hz stimulation may be suitable as a sham frequency to be used in the present study. This was only partly confirmed by the evaluation of the study results. Sham conditions could only be determined for the thermal detection, heat pain and pressure pain thresholds after 30 Hz-conditioning stimulation using the matrix array electrode. In further studies, it should be examined whether the sham conditions could also be achieved for MDT and MPT by varying stimulation frequencies. Probably, higher frequencies around or even above 50 Hz should also be studied, since other experiments showed that LFS leads to LTD using these frequencies [45], [46] and high frequency stimulation to LTP [47].

### Factor analysis QST Z-values

Following evaluation of the factor analysis (4 Hz-conditioning using the matrix electrode) QST parameters could be stratified to four major groups, namely the thermal and mechanical detection and pain thresholds. This finding points to differential effects of conditioning LFS to all relevant sensory pathways and partially may explain that a 30 Hz-conditioning frequency served as a sham frequency only for a minor proportion of QST parameters. Interestingly, factor 1 ‘mechanical pain’ and factor 2 ‘thermal pain’ showed similar groupings of the QST parameters included (Table 2, Fig. 7), although e.g. heat and cold pain are mediated through completely different peripheral nociceptive receptors at skin level. Accordingly, the effect of 4 Hz conditioning matrix electrode stimulation is more likely to be explained at a central level, such as the dorsal horn of the spinal cord.

### Limitations

It is a limitation of the present study that the number of subjects was small (n = 16). Moreover, using the matrix array electrode pin-skin resistance may slightly vary between the total number of 7×7 = 49 pins. Skin pores, hairs obstructing the pin-skin interface, the pin-skin contact-pressure as well as slight differences of skin moisture across the matrix area, may influence resistance. These factors will make it more difficult for the single constant current stimulator to maintain a constant current output controlling for the pin-skin potential difference dependent on the skin resistance. A 10% decrease on both skin conductivity and permittivity has been modelled in a recent study [48]. However, even if with a higher skin resistance for a couple of pins, adjacent pins will mediate current to the skin. And even though - most likely - current variations of smaller degrees will result comparing all pins, the strength of current applied is driven by the subjects’ pain ratings that allow controlling for the overall impression of the electrically evoked sensation.

The Finite Element Method (FEM) is just a theoretical model and can currently not be validated in a human model. It should also be noted that quantitative sensory testing is a subjective, psychophysical method that includes pre-defined stimuli activating different sensory pathways [49]–[51]. The evaluative plausibility and quality of the results and the repeatability and comparability depends on the understanding and the motivation of the subject. In the present study we examined evoked types of pain in healthy human subjects. Accordingly, our findings cannot be extrapolated to treatment effects addressing ongoing pain syndromes in chronic pain patients. Moreover, thermal QST was performed using a 3×3 cm contact thermode that may be disproportionate for the stimulation area of the small concentric electrode compared to the larger matrix array electrode.

## Conclusions

It was the main goal of the present study to examine the effects of a recently developed matrix array electrode compared to a conventional concentric electrode. Both types of electrodes were randomly assigned to a 4 Hz-conditioning vs. 30 Hz-conditioning stimulation using a double-blinded cross-over design in 16 healthy human subjects. It is the main finding of the present study that the matrix electrode better than the concentric electrode lead to mechanical hypoesthesia rather than hypoalgesia after low frequency 4 Hz-conditioning stimulation. Only the matrix electrode was able to reduce deep pain sensitivity as well, a finding that is consistent with the concept of a centrally mediated heterosynaptic LTD in the human nociceptive system. This finding has possible implications for further studies in patients suffering from chronic pain originating from deeper tissues.
